# Pathological Changes in Microvascular Morphology, Density, Size and Responses Following Comorbid Cerebral Injury

**DOI:** 10.3389/fnagi.2019.00047

**Published:** 2019-03-27

**Authors:** Zareen Amtul, Jun Yang, Ting-Yim Lee, David F. Cechetto

**Affiliations:** ^1^Department of Anatomy and Cell Biology, University of Western Ontario, London, ON, Canada; ^2^Robarts Research Institute, University of Western Ontario, London, ON, Canada

**Keywords:** beta-amyloid, ischemia, microvessels, basement membrane, vascular anatomy

## Abstract

Aberrations in brain microcirculation and the associated increase in blood-brain-barrier (BBB) permeability in addition to neuroinflammation and Aβ deposition observed in Alzheimer’s disease (AD) and ischemia have gained considerable attention recently. However, the role of microvascular homeostasis as a pathogenic substrate to disturbed microperfusion as well as an overlapping etiologic mechanism between AD and ischemia has not been thoroughly explored. In this study, we employ temporal histopathology of cerebral vasculature in a rat model of β-amyloid (Aβ) toxicity and endothelin-1 induced-ischemia (ET1) to investigate the panorama of cerebral pathology and the protein expression on d1, d7, and d28 post-injury. The combination of Aβ and ET1 pathological states leads to an alteration in microvascular anatomy, texture, diameter, density, and protein expression, in addition to disturbed vessel-matrix-connections, inter-compartmental water exchange and basement membrane profile within the lesion epicenter localized in the striatum of Aβ+ET1 brains compared to Aβ and ET1 rats. We conclude that the neural microvascular network, in addition to the neural tissue, is not only sensitive to structural deterioration but also serves as an underlying vascular etiology between ischemia and AD pathologies. Such investigation can provide prospects to appreciate the interrelationships between structure and responses of cerebral microvasculature and to provide a venue for vascular remodeling as a new treatment strategy.

## Introduction

In general, the microvascular bed of the brain is considered functionally adynamic and less sensitive to the morphological injuries than the neurons they serve and their supportive cells (del Zoppo et al., 2000), due to the comparative resistance of brain endothelium to hypoxia, to a certain extent (Tagaya et al., 1997). However, cerebral microvessels [includes the capillaries and their afferent (arterioles) – efferent (venules) connections] are either the target or consequence of numerous insults such as focal ischemia (del Zoppo et al., 2000), cerebral amyloid angiopathy (Ujiie et al., 2003), vascular dementia (Parnetti et al., 1994), hypertensive angiopathy (Baumbach and Heistad, 1989), autoimmune vasculitides, hyperglycemia, and inflammatory disorders (Paul et al., 2007). Chronic hydrocephalus, in particular, is associated with reduced cerebral blood flow and direct compression of fibers, blood vessels or brain tissue [*reviewed in* Owler and Pickard (2001)].

Consequential footprints of vascular defects are BBB breakdown, leakage of blood-borne molecules, swelling of astrocytic endfeet, interruption of inter-compartmental fluid exchange, disruption of basement membrane (BM), vessel-matrix-connections (Bailey et al., 2004), and disturbed capillary physiology (Paul et al., 2007; Brown and Thore, 2011) that leads to altered brain microcirculation [*reviewed in* Amtul and Hepburn (2014)]. Vascular functional defects have been described in AD transgenic animal models (Ujiie et al., 2003) and patients (Farrall and Wardlaw, 2009). For instance, vaccination against β-amyloid (Aβ); the hallmark of the AD, has been shown to reverse BBB pathology (Dickstein, 2006). Similarly, BBB breakdown due to the dissolution of primary endothelial cell permeability barrier and resulting hypoperfusion is also an antecedent event to cerebral ischemia (Hawkins, 2005). Intriguingly, there is a great majority of patients with first-ever stroke, who developed post-stroke dementia (Pendlebury and Rothwell, 2009), establishing that ischemic and AD pathogeneses interact with each other.

In this regard, we have demonstrated some very key pathological changes in the combined animal model of Aβ toxicity and ischemia, such as lateral-ventricle enlargement, neuroinflammation, Aβ deposition, altered insulin signaling/BBB, hippocampal injury, and impairment in learning and memory (Amtul and Hepburn, 2014; Amtul et al., 2011a,b, 2014, 2018a,c; Yang et al., 2014; Amtul, 2016, 2018). Using sequential computed tomography (CT) imaging we have also established altered microperfusion and associated elevated BBB disruption in the comorbid model of Aβ toxicity and ischemia (Yang et al., 2014; Amtul et al., 2018d). Surprisingly little is known about the effect of comorbid injury on the anatomical alteration of cerebrovasculature, topographic distribution, density, diameter and their role in maintaining cerebral water homeostasis and vessel-matrix-connections. Due to the serious consequences; damage to cerebral vasculature homeostasis and thus function must be considered a critical contributing component in the development of neurological disorders. Moreover, analyzing the vascular anatomical defects is also essential for adequate interpretation of brain imaging data to take us one step closer to develop remedies to restore vascular architecture, which may emerge as a new therapeutic target. Therefore, our working hypothesis is that the alteration in microvascular anatomy, texture, diameter, density, protein expression, and function serve as the interrelated pathogenic and etiologic link between AD and ischemia. Therefore, in the present study, we studied the anatomy of striatal microvasculature and the associated effects on water exchange and vascular-matrix connections by investigating various vascular markers at different time points in a comorbid rat model of Aβ toxicity and ischemia.

## Materials and Methods

### Study Design and Animal Treatment

All of the animal experiments were conducted in full compliance with the guidelines and approval of the Animal Care and Use Committee of the Western University (approval ID: 2008-113). All possible steps were taken to reduce the number of animals and their discomfort level. Two- to three-month old male Albino Wistar rats (Charles River Canada) weighing 250 to 310 g at the beginning of the experiment were used. Animals were divided into four groups for three different (d1, d7, and d28) timelines (*n* = 4 for each group). For stereotactic surgery, the animals were positioned into David Kopf stereotaxic apparatus (David Kopf Instruments, Tujunga, CA, United States) and anesthetized using 1.8% isoflurane. Body temperatures were maintained at 37°C throughout the surgery with the help of a heated blanket. To insert 30 gauge Hamilton needle, three burr holes were drilled in the parietal bone of each rat. All stereotaxic coordinates were determined using Paxinos and Watson Atlas (Paxinos and Watson, 2005).

### Induction of Striatal Cerebral Ischemia

The rat model of ischemia (group 1; ET1) receiving 60 pmol/3 μL injection of endothelin-1 (ET1; Sigma-Aldrich, Oakville, ON, Canada) into the right striatum (anterior/posterior +0.5 mm, medial/lateral -3.0 mm relative to bregma, and dorsoventral -5.0 mm below dura) has been described in detail elsewhere (Amtul et al., 2014, 2018a,b,c).

### Induction of β-Amyloid Toxicity

The rat model of β-amyloid toxicity (group 2; Aβ) receiving bilateral ICV injections of oligomeric 50 nM Aβ25–35/10 μL was modeled (anterior/posterior: -0.8 mm, mediolateral: ± 1.4 mm relative to bregma, and dorsoventral: -4.0 mm below dura) as described in detail elsewhere (Amtul et al., 2014, 2018a,b,c).

### Induction of Comorbidity and Control Procedure

Third group of rats received both bilateral ICV Aβ25–35 and unilateral striatal ET1 injections (group 3; Aβ+ET1) as described (Amtul et al., 2014, 2018a,b,c). The control rats (group 4; Control) received saline injections in identical locations.

### Post-surgery Treatment

After stitching the skin incisions, all rats were administered a subcutaneous injection of 0.03 mg/kg buprenorphine (Temgesic, RB Pharmaceuticals Ltd., Berkshire, United Kingdom) and an intramuscular injection of 1 mg/20 μl enrofloxacin antibiotic (Baytril, Bayer Inc., Canada). Twenty four hours (d1), 7 days (d7), and 28 days (d28) following surgery, corresponding rats were euthanized by an intraperitoneal pentobarbital overdose (Pentobarbital Sodique, Ceva Santé Animale, Cambridge, ON, Canada) followed by a transaortic perfusion with heparin containing phosphate buffered saline (PBS) and thereafter by 4% paraformaldehyde (pH 7.4).

### Tissue Preparation

The brains were harvested, postfixed in 4% paraformaldehyde, and cryopreserved in 30% cold (4°C) sucrose solution for 36 h. Next, the entire rat brains were serially sliced into 35 μm thick coronal cryosections.

### Immunohistochemistry

Immunoperoxidase protocol of avidin-biotin-peroxidase complex (ABC) was carried out as described (Hsu et al., 1981) to stain the free-floating cryosections with the following antibodies: Matrix metalloproteinase-9 (MMP-9; 1:1000, Millipore, AB19016), β-dystroglycan (βDG; 1:200, Leica B-DG-CE-S); βDG is an extracellular matrix adhesion protein abundant in astrocytic endfeet, co-localized with AQP4 and laminin and is needed for precise astrocytic anchoring around the cerebral vasculature (Zaccaria et al., 2001; Milner et al., 2008). SMI71 (Covance, 1:2000), an explicit endothelial barrier antigen (EBA) restricted to the luminal surfaces of all mature endothelium of the vasculature with intact BBB. BM-Laminin (1:1000, Sigma, L 9393) staining was used as an indicator of the BM rupturing, while neurons also contain laminin like molecules. For astrocytic antigen, glial fibrillary acidic protein (GFAP, 1:1000, Sigma, G3893) and binary water-channel protein, aquaporin4 (AQP4, 1:1000, Chemicon, AB2218) were employed. Fluorophore-conjugated donkey anti-rabbit FITC ( Sc-2090, 1:500) and donkey anti-mouse TR, (Sc-2785, 1:500) were prepared in 0.3% Triton X-100 containing 0.1 M PBS were used for fluorescence staining. Serums, biotinylated antibodies, and ABC reagent were purchased from the Vectastain Elite ABC Kit (Vector Laboratories, Inc., Burlingame, CA, United States).

### Analyses

Histological sections of brain were analyzed under light and fluorescence microscope. Leica Digital Camera DC 300 (Leica Microsystems Ltd., Heerbrugg, Switzerland) connected to a Leitz Diaplan microscope was used to take the photomicrographs. Cells and blood vessels were identified and counted, blindly, on the basis of positive labeling in the region of interest (ROI) on six non-neighboring slices with 210 μm distance between the sections using systematic random sampling method.

### The Region of Interest Determination

The region of positive laminin staining on consecutive slices in the right hemisphere (ipsilateral to the striatal ischemia) was noted as the location of the signal. This signal was limited to the striatal region between 1.6 to -0.92 mm anterior to posterior, relative to bregma. All histological analyses were performed in this region of the sections. The center of the ROI with dense laminin staining may alternatively be termed as lesion epicenter or lesion core, while the ROI surrounding the epicenter is termed as penumbra throughout the study. Six microscopic fields-of-view (each covering an area of about 0.50 mm^2^) in the ROI at 10× to 40× magnification with 0.25 to 0.65 numerical aperture were analyzed.

### Lumen Diameter Measurement

The diameters of lumen were determined using the ImageJ’s (National Institute of Health, version 1.48, United States) diameterJ plug-in analysis tool. Pixels in images with known scale bars were converted into micrometer in Image J. Next, a line selection was drawn across the structure of interest to measure the diameters.

### Vessel/Cell Density Measurement

For vascular/cellular density both manual as well as cell counter plugin was used to count the number of stained cells, vessels, layered vessels, split vessels, vessels with fibrosis and vessels with greater sizes. The results were presented as the number of vessels or cells per millimeter square of the ROI. Effect of MMP-9 on vascular fibrosis was quantified by calculating the ratio of the MMP-9 expression relative to the number of vessels displaying the fibrosis and expressed as negative log of n.

### AQP4 Polarization Measurement

AQP4 polarization is characterized as its dense, concentrated and localized expression within the astrocytic endfeet, ensheathing the BBB interface of cerebral microvessels, related to the astrocytic soma. Likewise, the loss of localized expression of AQP4 from perivascular endfeet processes and shifting toward astrocytic soma and coarse processes is referred to as its depolarization. AQP4 polarization was measured by counting the number of vessels expressing the AQP4 at their perivascular astrocytic BBB interface within each field of view. The relative values for AQP4 polarization on d7 and d28 were expressed as a percentage of the AQP4 polarization on d1 by normalizing it to 100 percent.

### Fluid-Filled Spaces Measurement

Fluid-filled spaces in the ROI were determined by drawing them on the computer screen with the help of image J quantification plugin analysis tools. The fluid-filled space on each section was normalized to the corresponding striatal volume, and thereafter to hemispheric swelling by dividing the contralateral hemisphere volume to the ipsilateral hemispheric volumes, multiplying by fluid-filled spaces. The relative area of fluid-filled spaces on d7 and d28 was calculated as the percentage volume of the fluid-filled spaces on d1 by normalizing it to 100 on d1.

### Statistical Analyses

All values were displayed as a mean ± standard error of the mean (S.E.M.). All measurements were analyzed by using one-way ANOVA followed by *post hoc* Dunnett’s tests. Except for inter-group comparisons at different time points, a *t*-test was used, and log values, two-way ANOVA was used. The significance level was *p* ≤ 0.05.

## Results

This is the first comprehensive histopathologic study of blood vessels pathology after the comorbid cerebral injury. These data suggest that the development of vascular pathology is a global phenomenon that affects the vascular dilation, lumen diameter, basement membrane integrity as well as the fluid homeostasis across the injured region after comorbid ischemia and amyloid toxicity.

### Dilated Blood Vessels and Lumen Diameter

**FIGURE 1:**
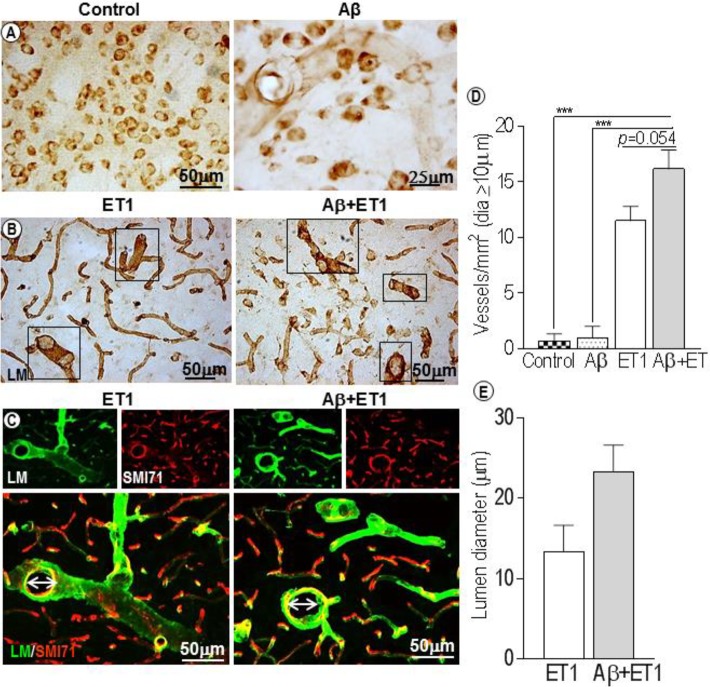
Dilated blood vessels and lumen diameter: Control and Aβ rats show BM-laminin positive neurons on d1. Aβ rats also show some isolated, inconsistent laminin stained microvessels **(A)**. Several dilated microvessels and numerous declining typical pyknotic neuronal cells can be seen in the ROI of ET1 and Aβ+ET1 rats on d1 **(B)**. Fluorescent staining of BM-laminin (green) indicates lumen diameters on d1 in the ROI or lesion core of ipsilateral striatum of ET1 and Aβ+ET1 rats. Punctate SMI71 staining (red) of endothelial cells can also be seen in the background. **(C)**. The plots show quantitative analyses of laminin-stained microvessels with equal or greater than 10 μm diameter in the ipsilateral striatum of control, Aβ, ET1, and Aβ+ET1 rats **(D)**, and lumen diameter of dilated microvessels in ET1 and Aβ+ET1 rats **(E)** on d1, ^∗∗∗^*p* < 0.001.

In control rats, laminin antibody only detected neurons, and in Aβ rats a few faint, isolated microvessels at each time point (Figure 1A). This is consistent with the literature that shows that in PFA-perfused tissues the laminin antibody does not stain BM-laminin but only neurons (Hagg et al., 1989). Conversely, a significantly robust network of capillaries containing BM-laminin is usually promptly evident after Aβ+ET1 or ET1 injections in the lesion epicenter due to the damage to BM and enhanced antibody penetration resulting in the robust staining of laminin protein. Alongside an intermittent yet widespread increase in the diameter of microvessels (square boxes) within the lesion core of Aβ+ET1 than ET1 rats (*p* = 0.054) was readily detected immediately after the injury compared to the control (*p* = 0.0005) and Aβ (*p* = 0.0006) rats (Figure 1B,D). Numerous thinning out laminin-positive pyknotic neurons were also obvious in the lesion core of ET1 and Aβ+ET1 rats on d1 (Figure 1B). Dilated microvessels in ET1 and Aβ+ET1 rats with larger lumen diameter (Figure 1C,E) also demonstrate punctate SMI71 staining of the luminal surface of the endothelial cells indicating BBB break down in those microvessels (Figure 1C).

### Basement Membrane Profile and Disruption

**FIGURE 2:**
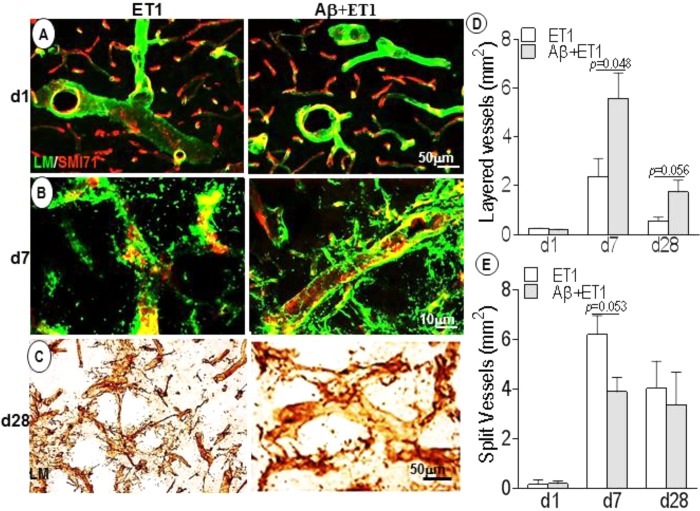
Basement membrane profile and disruption: Fluorescent images indicate an increase in BM-laminin immunoreactivity (green) in the lesion core localized to the ipsilateral striatum of ET1 and Aβ+ET1 rats from d1 **(A)** to d28 **(C)**. On d7, the BM starts splitting out in ET1 rats and layering out in Aβ+ET1 rats. A punctate and faint SMI71 staining indicative of disrupted BBB is also observed in microvessels with greater BM-laminin leakage, dissolution, and destabilization **(B)**. On d28, splitting reaches to its peak in ET1 rats while layering started fading out in Aβ+ET1 rats **(C)**. The plots show quantitative analyses of laminin-stained microvessels showing layering **(D)** and splitting **(E)** in the ipsilateral striatum of ET1 and Aβ+ET1 rats on d1, d7, and d28.

Basement membrane pathology including splitting, dissolution and rupturing was more prominent in ET1 rats compared to layering out tunica intima in Aβ+ET1 rats, that represents an earlier step to splitting (Figure 2A,B,D,E). On d28, laminin meshwork with degraded BM can be seen throughout the lesion core with more pronounced splitting around digested microvessels in ET1 rats and distinct layering in Aβ+ET1 rats (Figure 2C–E). Additionally, ET1 rats on d7 showed the splitting of BM-laminin with irregular SMI expression. In contrast, Aβ+ET1 rats showed layering of BM with relatively regular BBB (Figure 2B). Images on both fluorescent and 0.05% DAB staining were included to provide readers better clarity and resolution that might have been missed by the one procedure or another.

### MMP-9 Expression and Microvascular Fibrosis

**FIGURE 3:**
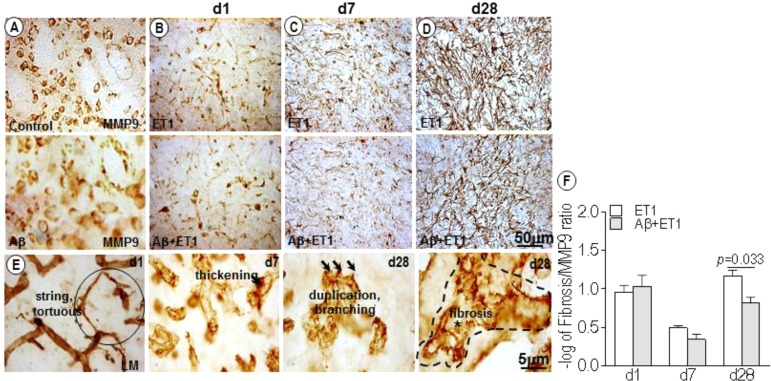
MMP-9 expression and microvascular fibrosis: DAB staining shows expression pattern of MMP-9 in the lesion core of the ipsilateral striatum of control, Aβ **(A)** ET1 and Aβ+ET1 rats from d1 to d28 **(B–D)**. In control and Aβ rats MMP-9 exclusively stain neuronal cells throughout the course of study **(A)**. On d1, MMP-9 is mainly expressed by the neuronal (pyknotic) cells and occasionally by the endothelial cells in the lesion core of ET1 and Aβ+ET1 rats **(B)**. On d7, MMP-9 staining still shows neuronal profile and appearance of astrocytic morphology **(C)**. On d28, MMP-9 acquires massive astrocytes appearance **(D)**. High-resolution images of BM-laminin staining show different stages of capillary wall pathology from d1 to d28 in Aβ+ET1 rats, such as stringed and tortuous structures (circle), local irregular thickening on the abluminal surface of capillaries, duplication, branching (three arrows), and fibrosis of BM (dotted line) **(E)**. The plot shows –log of the ratio of the MMP-9 expression relative to the number of vessels displaying fibrosis **(F)** in the ipsilateral striatum of ET1 and Aβ+ET1 rats on d1, d7, and d28.

In control and Aβ rats, MMP-9 appeared to stain a large number of neuronal cells, as obvious from their structure and distribution pattern (Figure 3A). While in ET1 and Aβ+ET1 rats on d1, MMP-9 stained striatal cells that appeared to be the reminiscent of microvascular endothelium, representing the disturbed vascular-matrix connection, with more in ET1 and less in Aβ+ET1 rats (Figure 3B). On d7, in the penumbra-core interface MMP-9 started appearing as astrocytes (Figure 3C). On d28 MMP-9 staining showed complete loss of endothelial profile observed on d1, and strongly indicated an expression of astrocytes in the lesion core with significantly more in ET1 brains than Aβ+ET1 (*p* < 0.05) rats (Figure 3D). Besides, the capillary structure in the lesion core started getting severely compromised (Figure 3E). The percent fibrosis of vessels in Aβ+ET1 rats was strongly correlated with an MMP-9 increase (*p* = 0.033) in the lesion core, suggesting that fibrosis was a critical process in the penumbra of Aβ+ET1 rats. The images themselves contain sufficient detail to clearly display capillary structures, allowing the visualization of principal BM layer and the vasculature. More detailed visualization of capillary atrophy intercalated by a dense BM network included string, fragmented and tortuous structures (circle). An uneven thickening on the abluminal surface of capillaries, duplication, branching (three arrows), and microvascular fibrosis of BM (dotted line), was common in the lesion core of treated rats, in general.

### AQP4 Polarization vs. Fluid Homeostasis

**FIGURE 4:**
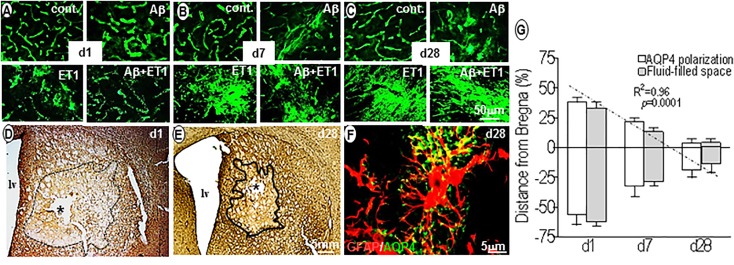
Aquaporin4 polarization vs. fluid homeostasis: Fluorescent images show the expression pattern of AQP4 immunostaining in the ROI of control, Aβ, ET1, and Aβ+ET1 rats from d1 to d28 **(A–C)**. The punctate AQP4 expression on d1 in ET1 and Aβ+ET1 rats **(A)** starting to depolarize on d7 **(B)** and intensely depolarized on d28 **(C)**. While control rats show smooth, regular AQP4 staining throughout, and Aβ rats show an increase in AQP4 haziness from d1 to d28 **(A–C)**. Low-resolution images show the topographical distribution of fluid-filled spaces by astrocytes **(D)** and βDG **(E)** immunostaining in the penumbra and lesion core; ROI of Aβ+ET1 rat on d1 **(D)** and d28 **(E)**. The epicenter of the lesion core also shows a prominent necrotic lesion (asterisk) **(D,E)**, lv; lateral ventricles. High-resolution image shows depolarization of AQP4 expression from GFAP positive astrocytic end-feet to the astrocytic soma **(F)**. The plot shows a quantitative decrease in AQP4 polarization and fluid-filled spaces in an anterior (+) to posterior (-) manner from the bregma (0), in the ipsilateral striatum of ET1 and Aβ+ET1 rats from d1 to d28 **(G)**.

Within 24 h, AQP4 staining was highly polarized around endothelial cells of microvessels in control (*p* = 0.002) and Aβ (*p* = 0.001) brains compared to the ET1 and Aβ+ET1 brains. On the contrary, AQP4 staining appeared punctate in the lesion core of ET1 and Aβ+ET1 rats. While the neuropil space in between the AQP4 stained vessels was missing any AQP4 staining in Aβ+ET1 rats, in ET1 rats it exhibited haziness indicative of relocation of AQP4 protein (Figure 4A). On d7, the depolarization of AQP4 became more distinct in ET1 and Aβ+ET1 rats and a hazy fluorescence appeared to extend into the neighboring neuropil compared to control and Aβ rats (Figure 4B). On d28, an increased AQP4 immunoreactivity was accompanied by the re-distribution of polarized AQP4 expression from astrocytic endfeet to the soma with more in ET1 rats than Aβ+ET1 rats (Figure 4C). A subset of microvessels in Aβ rats showed AQP4 haziness and depolarization on d28; however, it did not reach statistical significance (Figure 4C). Low-resolution images indicated the topographical immunostaining of GFAP (Figure 4D), and βDG positive (Figure 4E) astrocytes in the ipsilateral striatum of Aβ+ET1 rat brain on d28. On d1, both ET1 and Aβ+ET1 rats had approximately a100% increase in fluid-filled spaces in the ROI compared to control and Aβ rats, consistent with the vasogenic edema formation (Figure 4D,G). While, on d28 with the increase in AQP4 depolarization, the size of these fluid-filled spaces got shrunk to one-third of their original size from d1 (*R* = 0.96, *p* = 0.0001) (Figure 4E). The dark line showed the division between core and penumbra, where a palisade layer is formed by GFAP positive astrocytes. While βDG expression on astrocytic end feet demonstrates an attempt by the astrocytes to restore vascular-matrix connections (Figure 4D,E). The high-resolution image showed co-labeling of AQP4 and GFAP staining to demonstrate AQP4 depolarization from astrocytic foot processes to soma in the fluid-filled space in an Aβ+ET1 rat on d28, in an attempt to increase the inter-compartmental water exchange from fluid-filled spaces to the bloodstreams (Figure 4F). Co-expressed GFAP and AQP4 staining were not used for any quantitative analyses.

## Discussion

In this study, we demonstrate that the comorbid occurrence of ischemia and Aβ toxicity provoked substantial dynamic, rapid and highly coordinated changes in the vascular anatomy and physiology in the striatal lesion core and penumbra of Aβ+ET1 rats. The present study also showed a correlation between vascular structure deterioration and the deficits in microvascular responses, such as inter-compartmental water exchange and vessel-matrix-connections of cerebral capillaries after comorbid injury.

Immediately after ET1 and/or Aβ+ET1 injections, the compression of small capillaries (not arterioles and venules) and a drop in cerebral perfusion, due to the potent vasoconstrictive effects of ET1, led to the dilation of precapillary resistance vessels in order to maintain cerebral perfusion. This was observed by the homogenous distribution of certain vessels with increased diameter throughout the penumbra in Aβ+ET1 rats compared to ET1 rats on d1. This shifted the calculated average size of vessels to the considerably larger values in Aβ+ET1 rats. Whereas the changes in the microvessels structure in ET1 rats accompanied by a decrease in the outer diameters of the vessel lumen. These alterations perhaps referring to some hyperplasia or hypertrophy of the vessel wall in these rats, significantly impacted vascular function (Baumbach and Heistad, 1989). This also referred to a different pattern of remodeling in Aβ+ET1 rats compared to ET1 rats alone (Figure 1).

Following flow restoration in the constricted vessels, the downstream capillary network endured the impact (del Zoppo et al., 2000). For instance, in injured rats once maximum vasodilation was reached, autoregulation failed and progressive continued compression of small capillaries (Liebeskind, 2003) finally resulted in the opening of tight junction proteins of endothelial cells and breakdown of BBB permeability, as observed by a punctate EBA or SMI71 staining in ET1 and Aβ+ET1 rats on d7 (Figure 2B).

A breakdown of BBB or permeability barrier was accompanied by significant alterations in microvessel structure in these rats. As, spatially endothelial cells are in a perfect position to sense minor changes in cerebral perfusion, thus shear stress. These pathologic changes seemed to first affect the intima, media, and adventitia layers of microvessels. Immediately after injury, these layers started separating from the vessels, however, more robustly in ET1 rats compared to the Aβ+ET1 rats. As, ET1 rats demonstrated splitting, which is usually followed by a vascular layering of tunica intima; that comprised of endothelial cells and the contiguous BM (Stratman et al., 2009), starting from d7 (Figure 2B). Possibly, our endpoint timing of euthanization couldn’t catch the window of vascular layering in ET1 rats. Splitting, in turn, led to multiple tears within the microvascular layers and BM-endothelium of ET1 rats by d28 (Figure 2C).

This study also highlights vascular fibrosis or vascular BM thickening as a pathologic phenomenon associated with ischemia and comorbid injury. In Aβ+ET1 brains, BM sufferd the most dramatic and pronounced damage and emerged as a central target of vascular pathological changes (Hawkes et al., 2011; Figure 3). Vascular fibrosis; linked with many pathological processes and clinical conditions (Harvey et al., 2016) is characterized by reduced compliance, such as lumen diameter, increased vascular collagen, reduced elasticity (Selvin et al., 2010), and associated excessive deposition/remodeling of extracellular matrix (ECM) (Iwazu et al., 2011). Increased expression and activation of matrix metalloproteinases (MMP) is characterized as one of the molecular mechanisms underlying ECM remodeling and vascular fibrosis (Epstein et al., 1994). Accordingly, many of the partially digested microvessels that still remained in the striatal lesion core were associated with MMP-9 positive staining in ET1 and Aβ+ET1 rats on d1 (Figure 3B) indicating the diminishing or residual vessel-matrix-connections. By d28 complex interplay between ischemia, Aβ toxicity and astrocytic expression of MMP-9 (Zhao et al., 2006; Wang and Hatton, 2009; Figure 3D), resulted in accelerated vascular remodeling (as observed by elevated astrocytic expression) in ET1 rats and fibrosis (as accompanied by lowered astrocytic expression) in Aβ+ET1 rats. This again confirms a different pattern of remodeling in ET1 and Aβ+ET1 rats, as mentioned above. In addition, the presence of cerebral microvessels with string, tortuous structures (Figure 3E) and reduced capillary density in Aβ+ET1 rats may be responsible for the increased resistance of microvessels and resulting moderate hemodynamic obstruction in these rats by d28, reported earlier (Yang et al., 2014).

The very peculiar set of idiosyncratic variations was noticed in the polarized expression of end feet pool of AQP4 from d1 to d28 (Figure 4). Within 24 h, punctate, granulated, polarized expression of AQP4 channel protein, enclosing the cerebral endothelial layers in the lesion core demonstrated re-localization or redistribution from the end feet membranes to the parenchymal soma membranes of the astrocyte body starting from d7 (Wolburg-Buchholz et al., 2009). Perhaps this is to aid in astroglial migration toward fluid-filled spaces in forming glial scar in the lesion core, necessary for filopodia formation (Saadoun, 2005; Badaut et al., 2007), as well as to help in intra-astroglial water accumulation or cytotoxic edema in an effort to clear up fluid-filled spaces formed in the lesion core of ET1 and Aβ+ET1 rats on d1 (Figure 4D,E). On d28, the reduction in fluid-filled spaces in injured rats when AQP4 depolarization was at its peak, confirmed its role as the principle bidirectional water transporting channel of astrocytes (Nagelhus et al., 2004) and thus in fluid clearance from the brain into the bloodstream.

Currently, it is uncertain if Aβ rats will express significant degeneration in vascular physiology and markers in a four-week time frame, as Aβ toxicity alone was not detrimental enough. Low numbers of animals per group (*n* = 4) might be a contributing limiting factor. However, in the given time, striatal microvessels in the Aβ rats showed vascular degeneration and BBB disruption compared with controls, although non-significantly. Further studies with extended time intervals will likely provide an enhanced appreciation of the structural links of vascular injury with vascular responses in these rats.

In summary, we propose that the anatomical or morphological alterations in the cerebral network of microvessels after injury could be used effectively to describe the neuropathology of the injured brain. For example, the impaired BBB restoration after Aβ+ET1 toxicity reported earlier (Amtul et al., 2018d) might be the direct cause of the altered anatomy of the cerebral vasculature in these rats. Lastly, longitudinal magnetic resonance evaluations can aid to correlate imaging of vessel size and microvascular morphology to the fate of neural tissue to further improve the tailoring of the treatments. Such an investigation could lead to a new rationale to induce vascular remodeling by designing therapeutics for demented patients.

## Author Contributions

The manuscript was written through the contributions of all authors. All authors have given approval to the final version of the manuscript.

## Conflict of Interest Statement

The authors declare that the research was conducted in the absence of any commercial or financial relationships that could be construed as a potential conflict of interest.

## Abbreviations

ABC: avidin-biotin complex
AD: Alzheimer’s disease
APP: amyloid precursor protein
AQP4: aquaporin4
Aβ: β-amyloid
BBB: blood-brain barrier
DAB: 3,3′-diaminobenzidine tetrahydrochloride
ET1: endothelin-1
ICV: Intracerebroventricular
MMP-9: matrix metalloproteinase9
PBS: phosphate-buffered saline
βDG: β-dystroglycan.
